# Making sense of glucose sensors in end-stage kidney disease: A review

**DOI:** 10.3389/fcdhc.2022.1025328

**Published:** 2022-12-19

**Authors:** Mark E. Williams, Devin Steenkamp, Howard Wolpert

**Affiliations:** ^1^ Renal Unit, Joslin Diabetes Center, Boston MA, United States; ^2^ Section of Endocrinology, Diabetes, and Nutrition, Department of Medicine, Boston Medical Center, Boston, MA, United States; ^3^ Boston University School of Medicine, Boston, MA, United States

**Keywords:** Diabetes, glucose sensors, hemodialysis, glycemic control, complications

## Abstract

Diabetes mellitus remains the leading cause of end-stage kidney disease worldwide. Inadequate glucose monitoring has been identified as one of the gaps in care for hemodialysis patients with diabetes, and lack of reliable methods to assess glycemia has contributed to uncertainty regarding the benefit of glycemic control in these individuals. Hemoglobin A1c, the standard metric to evaluate glycemic control, is inaccurate in patients with kidney failure, and does not capture the full range of glucose values for patients with diabetes. Recent advances in continuous glucose monitoring have established this technology as the new gold standard for glucose management in diabetes. Glucose fluctuations are uniquely challenging in patients dependent on intermittent hemodialysis, and lead to clinically significant glycemic variability. This review evaluates continuous glucose monitoring technology, its validity in the setting of kidney failure, and interpretation of glucose monitoring results for the nephrologist. Continuous glucose monitoring targets for patients on dialysis have yet to be established. While continuous glucose monitoring provides a more complete picture of the glycemic profile than hemoglobin A1c and can mitigate high-risk hypoglycemia and hyperglycemia in the context of the hemodialysis procedure itself, whether the technology can improve clinical outcomes merits further investigation.

## Introduction

The world-wide incidence and prevalence of end-stage kidney disease (ESKD) have been increasing annually, with type 2 diabetes mellitus the leading cause of ESKD in most developed countries (1) . The global prevalence of diabetes in the ESKD population increased from 19.0% in 2000 to 29.7% in 2015, and the incident population from 22.1% to 31% (2).

Insufficient glucose monitoring has been identified as one of the gaps in care for hemodialysis patients with diabetes (3, 4). Both a previous American Diabetes Association Diabetic Kidney Disease Consensus Conference report (5) and the recent 2020 KDIGO Clinical Practice Guideline (6) have highlighted the importance of meeting recommended glucose targets. However, the benefit of glycemic control in patients with diabetes and ESKD continues to be debated (7, 8), and there is uncertainty regarding appropriate glucose targets. In the ESKD population, the lack of reliable methods to assess glycemia has hindered the attainment of optimal glycemic control (9).

The recent American Diabetes Association guidelines have highlighted the increasingly important role of continuous glucose monitoring (CGM) in glycemic management in the general diabetes population (10, 11). A recent consensus conference has recommended that CGM training programs be expanded to all healthcare professionals involved in multidisciplinary diabetes management (12). The focus of this review is to evaluate the increasing role of CGM in diabetic ESKD.

## Glucose homeostasis in ESRD

Type 2 diabetes constitutes about 90% of all cases of diabetes, and results from tissue insulin resistance with impaired peripheral glucose uptake and dysregulated hepatic glucose production, and progressive pancreatic beta-cell dysfunction (13, 14). The development of chronic kidney disease (CKD) often alters the typical natural history of diabetes. Diabetes physiology in the ESKD patient is different and more complex than in the general diabetes population, with significant alterations of both glucose and insulin homeostasis (15). The result may be wide glycemic excursions with frequent hypoglycemia and hyperglycemia (16). Factors which contribute to the complex abnormalities in glucose homeostasis include abnormal insulin metabolism with reduced renal insulin clearance, increased insulin resistance, and the effects of the dialysis procedure.

## Assessment of glycemic control in ESKD

**Figure 1:**
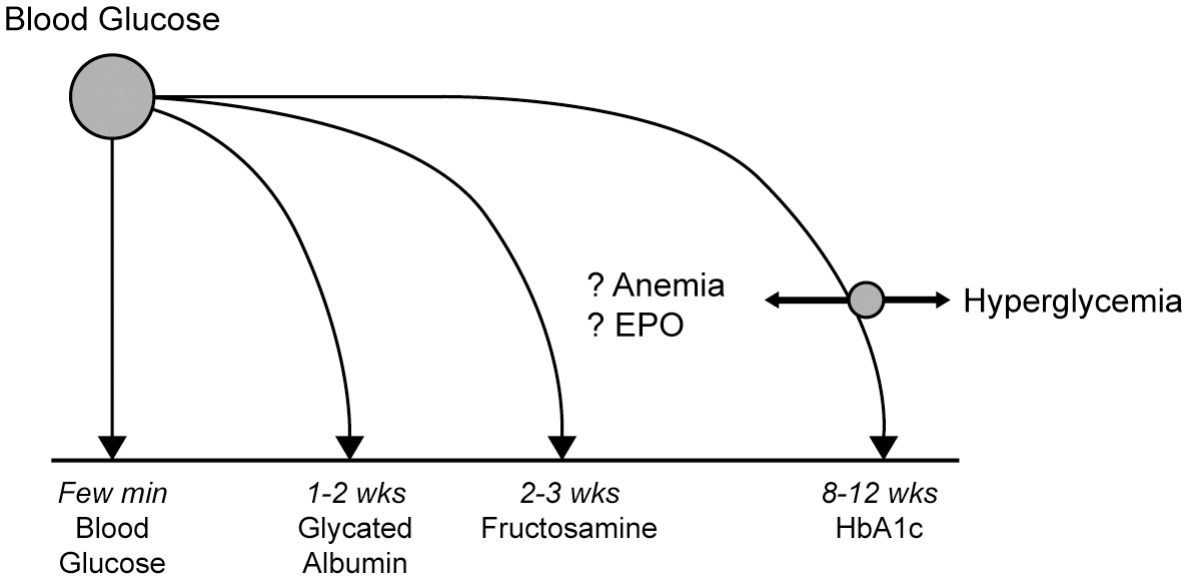
Measures for assessing glycemic control in ESKD patients with diabetes (17).

Historically, the assessment of glycemic control has relied upon two categories of testing: serum biomarkers (hemoglobin A1c [HbA1c], glycated albumin, fructosamine), and capillary blood glucose measurement (self-monitoring of blood glucose [SBGM]) (17) (**Figure 1**).

Long-term glycemic markers have limitations in end-stage kidney disease (18). The NKF-KDOQI guidelines for diabetic CKD have acknowledged that there is a deficiency in data for validating markers in patients with reduced kidney function (KDOQI) (19). As reported by the recent Kidney Disease: Improving Global Outcomes (KDIGO) guidelines, HbA1c is known to be inaccurate in patients with advanced kidney impairment (3). In the general diabetes population, a wide range of mean glucose concentrations is possible for any HbA1c value, which has been termed “the fallacy of average” (20). Furthermore, HbA1c measurements tend to run lower in advanced CKD with resultant under-estimation of hyperglycemia (15). Factors contributing to lower HbA1c values in ESKD include reduced erythrocyte lifespan/time available for glycosylation of hemoglobin, anemia, and exposure to erythropoiesis-stimulating agents (21, 22).

The role of tight glycemic control in improving clinical outcomes in ESKD remains controversial (23, 24). Using different retrospective ESKD database analyses, Kalantar-Zadeh (9) and Williams (8) found that glycemia, when considered as a continuous variable, was associated with poorer survival only at extremes of HbA1c. The optimal target HbA1c range for patients with diabetes on dialysis therefore differs from that of the general diabetes population, i.e. <7% (25). The Kidney Disease Outcome Quality Initiative (KDOQI) Clinical Practice Guidelines for Diabetes have highlighted the higher mortality risk in hemodialysis patients with HbA1c levels <6.5 or >8.0% (9). The 2020 KDIGO guidelines indicate that glycemic control must be individualized, with tighter (HbA1c < 7%) or looser (HbA1c > 8%) targets based on comorbidities, risk of hypoglycemia, life expectancy, and other factors (3, 22).

Alternative measures to HbA1c, including glycated albumin and fructosamine, have been proposed for the ESKD population. Data from Freedman et al. suggested that increasing concentrations of glycated albumin, but not of HbA1c, predicted hospitalization and survival in patients with diabetes undergoing dialysis (26). Some recent studies have shown an association between glycated albumin levels and one-year mortality, and suggested potential superiority over HbA1c (27, 28). Limited evidence suggests that the target range for glycated albumin in ESKD patients should also exceed the reference range (27).

HbA1c does not capture the full range of glucose values in patients with diabetes. Alternative established glucose metrics, including patterns of hyper/hypoglycemia and glucose variability, are known to contribute to clinical outcomes in the general diabetes population (20, 29–31). Patients with diabetes and ESKD frequently experience wide glycemic excursions, with both hyperglycemia and hypoglycemia (16). Frequent SMBG has been regarded as an essential element of diabetes management and, until the introduction of CGM, was the cornerstone of intensive diabetes management regimens. Fingerstick SMBG is associated with improvements in HbA1c, with reduced hyperglycemic excursions and rates of hypoglycemia (32). However, SBGM is limited by patient inconvenience and potential discomfort, cost, and variable device accuracy (33).

## Continuous glucose monitoring

CGM has emerged as an innovative and important alternative to SBGM in diabetes management (12, 34). CGM devices measure the interstitial glucose concentration every 1-15 minutes (frequency varies depending on device type), providing a more comprehensive picture of postprandial glucose excursion and also unmasking hypoglycemia particularly in the overnight period (35). CGM devices generate a set of metrics that are valuable in assessment of glucose control, and can be used in clinical practice to complement the HbA1c measurement (36–42) (see below). Multiple subsequent randomized clinical trials have now shown unequivocal improvement in hemoglobin A1c with reduction in hypoglycemia with use of CGM by individuals with type 1 and type 2 diabetes (10, 35, 43–47). Improvements in the advances in the accuracy and ease-of-use of CGM devices have established this technology as a replacement for fingerstick SMBG and as the new gold standard in glucose monitoring (48–51).

## The hemodialysis treatment

### Effect of the hemodialysis treatment on glucose levels

It is widely recognized that plasma glucose levels decrease significantly during the hemodialysis procedure. Sudha et all measured capillary blood glucose levels, just before dialysis and 2 hours after dialysis, and compared with fasting and postprandial glucose levels on–off dialysis days (52). There was a significant (35.8%) decrease in blood glucose levels two hours after dialysis in comparison to predialysis levels (from mean level of 258mg/dL/14.3 mmol/L to 165 mg/dL/9.2 mmol/L). Abe et al. evaluated changes in plasma glucose levels specifically between pre- and post-dialyzer sites during hemodialysis (53). Results confirmed removal of glucose by the dialysis procedure. In hemodialysis patients with diabetes, CGM data indicate that on dialysis days mean glucose levels are lower and that glucose fluctuations, as indicated by standard deviation and mean amplitude glycemic excursion (MAGE) are greater than on days without hemodialysis (54). A component of glucose homeostasis unique to the hemodialysis procedure is the presence of dextrose, chemically identical to glucose, as a constituent of the dialysate fluid (6, 55). Addition of dextrose protects from severe hypoglycemia.

### Effect of the hemodialysis treatment on insulin

The hemodialysis procedure is known to result in removal of circulating insulin while at the same time improving insulin sensitivity. An understanding of the impact of these effects underscores the potential value of CGM in the chronic hemodialysis setting.

#### Insulin clearance

For patients requiring hemodialysis, insulin is frequently administered prior to the hemodialysis session. Administered insulin contributes to the intradialytic decrease in plasma glucose level noted above, but circulating insulin is removed by hemodialysis so that blood insulin levels decrease (53). The molecular size of circulating plasma insulin monomer (5.8kDa) would indicate that filtration through the membrane would contribute to its removal. Adsorption of insulin onto the dialyzer membrane is an additional factor (56).

#### Insulin sensitivity

Tissue insensitivity to insulin is a common feature of ESKD, especially in individuals with type 2 diabetes (57, 58). The mechanism remains unclear, but contributing factors may involve circulating pro-inflammatory cytokines (59). Skeletal muscle is the primary site accounting for insulin resistance in patients with uremia (60). Uremic insulin resistance is known to improve after initiation of chronic renal replacement therapy (61, 62). Using a modified euglycemic clamp methodology Sobngwi et al, evaluated the insulin requirements necessary to achieve euglycemia in the pre-, intra-, and post-hemodialysis period and noted a 25% lower exogenous insulin requirement immediately after the dialysis procedure (62).

#### Net effect

**Figure 2:**
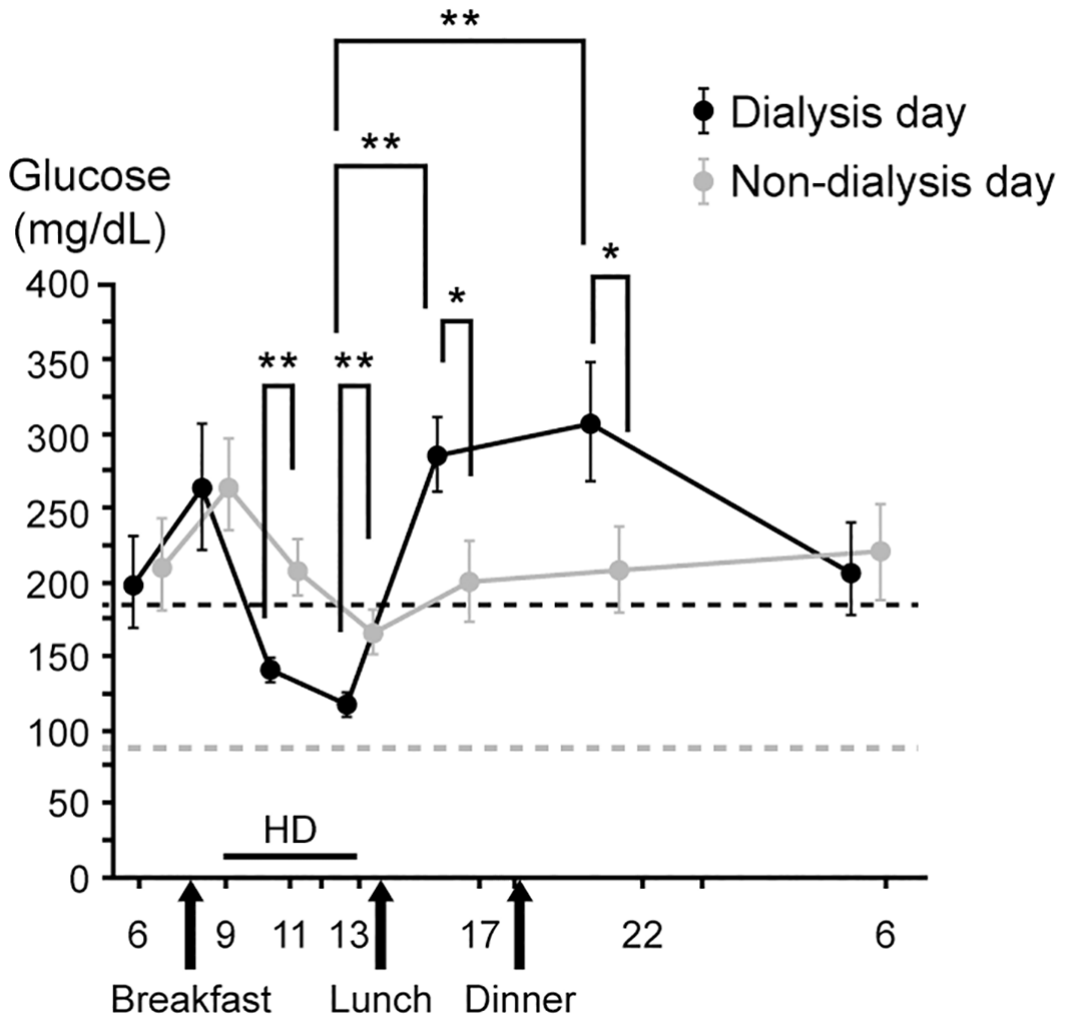
Circadian changes in plasma glucose level in hemodialysis patients with diabetes on dialysis day (dark line) and non-dialysis day (light line). *P<0.05; **P<0.01. (Adapted with permission from (63). HD, hemodialysis; SD, standard deviation).

The net effect is to make diabetes management less predictable in the ESKD patient. In general, clinical research studies suggest a prototypic peri-dialytic paradigm of hypoglycemia followed by rebound hyperglycemia shown in **Figure 2** (63). In addition, circadian changes in plasma glucose levels may differ between dialysis and non-dialysis days.

Of clinical relevance, the signs and symptoms of hyperglycemia will be modified, with increased thirst, fluid overload, and hyperkalemia expected but not polyuria or volume contraction. Hemodialysis patients, particularly with type 2 diabetes, rarely develop diabetic ketoacidosis (63). Development of hypoglycemia unawareness due to recurrent hypoglycemia (64) is an additional risk in the ESKD patient on dialysis. Hypoglycemia has recently been associated with the occurrence of cardiac arrhythmias in patients with diabetes and CKD (65).

#### Glycemic variability

**Figure 3:**
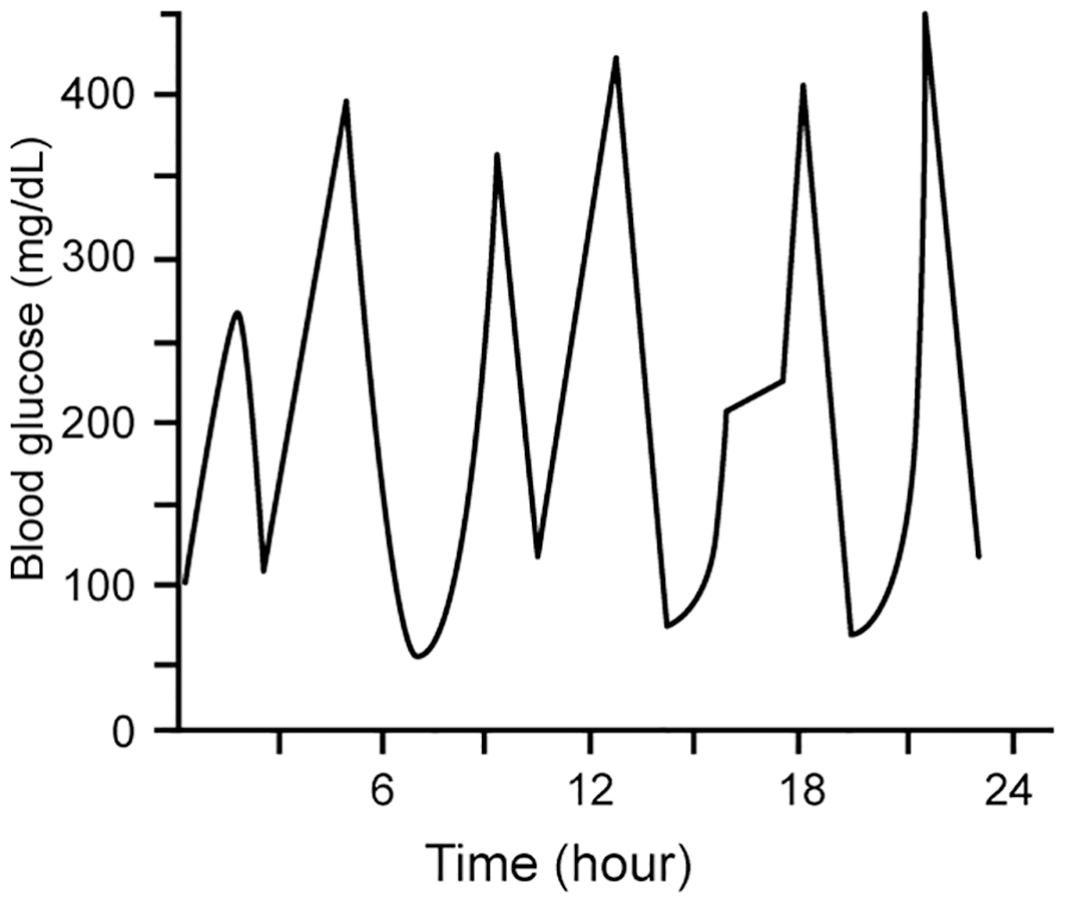
Short-term glycemic variability, represented by within-day measures of blood glucose as determined by SBGM and CGM. Modified with permission from 68).

ESKD patients with diabetes are known to have greater fluctuations than dialysis patients without diabetes (54). Glycemic variability – i.e., fluctuations of measures of glycemic control over a given interval of time - has been termed the “third component of dysglycemia” in diabetes (66). Accumulating data suggest that glycemic variability is an independent risk factor for diabetes complications (67, 68). Glycemic variability may be evident in both long-term measures (serial determinations of HbA1c), or in short-term measures (based on serial measures using CGM or blood glucose measurements), as shown in **Figure 3** (68). Both glucose and HbA1c variability have been shown to predict hospitalization risk in ESKD (69).

## Interstitial fluid and its compostion

CGM devices measure interstitial fluid glucose. The interstitial compartment comprises approximately eighty per cent of extracellular water, the remainder comprising the plasma compartment (70). The main solute constituents (excluding glucose) of these body fluid compartments are electrolytes (71). Interstitial fluid homeostasis is considered to be regulated at the local tissue level. Fluid exchange homeostasis between the plasma and interstitium is governed by the interaction of key components of Starling’s hypothesis, such as the interstitial hydrostatic pressure (72). The pressure/volume status of the interstitium is determined by a complex interaction involving fluid inflow (blood capillary filtration), fluid outflow (lymphatics), and the interstitial compartment’s tissue compliance (73). Interstitial fluid pressures are slightly negative in healthy controls, and positive in CKD patients with edema (74). This increase in interstitial fluid pressure in CKD would be expected to result in reduced transcapillary filtration into the interstitium, preventing further fluid influx, and/or increased unidirectional outward lymphatic flow (73). Glucose dynamics between the plasma and interstitial fluid may be considered a “two-compartment” model (75). Glucose diffuses from the capillary endothelium to the interstitial compartment (76), driven by the glucose concentration gradient across capillaries, with a time lag in the passage of glucose from blood to the interstitium (77).

In individuals with kidney impairment, extracellular fluid volume, interstitial fluid volume and interstitial pressure are significantly elevated (73, 74). It is likely that there is distortion in glucose homeostasis caused by nonequilibrium conditions between the vascular compartment and the extracellular compartment (57). Although the time lag in the distribution of glucose from blood to the interstitium under normal conditions has been investigated (77, 78), changes during the hemodialysis procedure are uncertain.

## Glucose sensor technology

In recent years,advances in sensor technology have set the stage for more widespread adoption of CGM into diabetes care (79). Among Current FDA-approved CGM devices are the Abbott Freestyle Libre (Abbott Diabetes Care, Alameda CA), Dexcom G6 (Dexcom, San Diego CA) and Medtronic Guardian (Medtronic, Northridge CA) CGM devices. These devices typically consist of a transcutaneous sensor probe that is inserted into subcutaneous tissue and measures glucose concentrations in the interstitial fluid; the glucose measurement technology in these sensor probes is electrochemical and utilizes the enzyme glucose oxidase, which generates hydrogen peroxide and an electron flow proportional to the glucose concentration. Because of the specificity of glucose oxidase for beta-D-glucose, these sensors are not expected to be subject to interference by other monosaccharides and polysaccharides [80. However, acetaminophen at doses in excess of 1gram every 6 hours and high dose ascorbic acid can affect performance of the Abbott Freestyle Libre (81). Local skin reactions from adhesive tape that secures the transmitter to the skin can occur (82, 83). Another approved device, the Eversense CGM (Senseonics, Germantown MD) is clinician-implanted, with need for an office-based procedure for insertion/removal every 90 days (84), and functions non-enzymatically, utilizing a hydrogel that fluoresces on glucose binding. Studies indicate that mannitol and tetracycline can interfere with its accuracy (85).

Traditionally, CGM devices required that the patients perform fingerstick capillary blood glucose measurements for calibration (86). However, the Abbott Freestyle Libre and Dexcom G6 are factory-calibrated, and there is no need for regular calibration to ensure CGM accuracy.

### Evaluation of CGM accuracy

**Figure 4:**
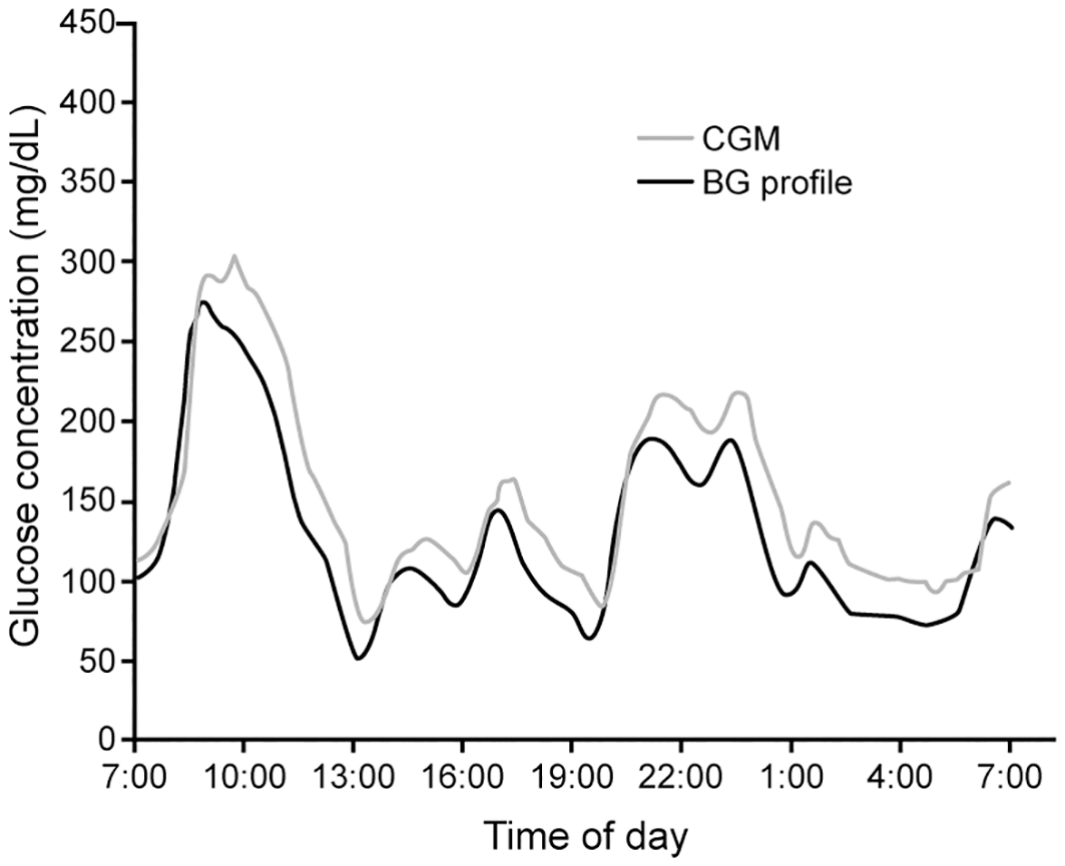
Measured CGM data and paired blood glucose measurements (BG profile) in a patient with diabetes (Adapted with permission from Ref (87).

The measurement accuracy of CGM devices is assessed using plasma glucose as the reference standard (**Figure 4**). The mean absolute relative difference (MARD) between CGM measurements of and matched reference values is the commonly used metric to evaluate device accuracy. A MARD ¾ 10% is widely cited as the desired goal in CGM device accuracy (30, 88). However, it is noteworthy that many of the studies demonstrating the clinical benefits of CGM were conducted with devices with MARD that did not meet this cut-point (35, 89–91).

**Figure 5:**
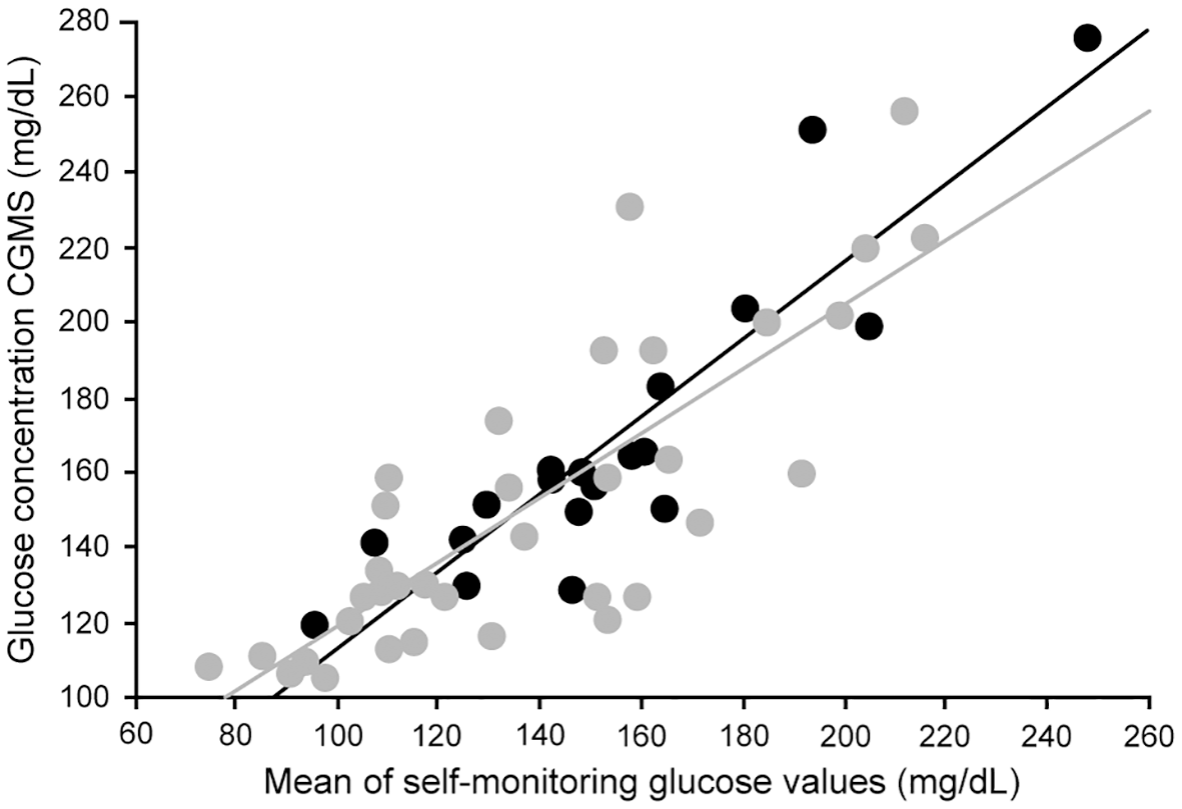
Correlation between mean glucose levels obtained through glucose meters and CGM in hemodialysis type 2 diabetes (dark circles) and nondialysis type 2 diabetes (light circles) (Ref (94).). Correlation in the entire patient group (r = 0.84; P<0.00001). Correlation in hemodialysis type 2 patients (dark line) (r = 0.90; P<0.0001). Correlation in nondialysis type 2 patients (light line) (r = 0.81; P<0.0001).

Research has revealed that paired glucose concentrations in the capillary and venous compartments and the interstitial space will differ when the glucose is changing rapidly (78). Because of this, apparent accuracy of CGM devices tends to be lower in individuals with high glucose variability and at the extremes of the glucose range (92, 93). This is an important consideration in the evaluation of studies of device accuracy. In patients with chronic kidney disease, data correlating CGM and plasma glucose to validate the accuracy of CGM are limited. Riveline evaluated the relationship between CGM and blood glucose measurements in nineteen hemodialysis patients on hemodialysis studied over four days (94). There was a high correlation between mean values using the two techniques (**Figure 5**), and the correlations were similar in dialysis and non-dialysis patients.

Mambelli et al. found very good accuracy of the Abbott Libre CGM in patients with type 2 diabetes on hemodialysis, with 97% of measurements in A+B regions of the Clarke error grid (95). In contrast, another study in patients with type 2 diabetes on hemodialysis by Yajima et al. showed poorer accuracy with the Abbott Libre CGM (96). The accuracy in patients on hemodialysis is not affected by hydration status measured *via* impedance spectroscopy (97). A small study in twelve patients (98) showed better MARD at the beginning of the dialysis treatment (r=0.731) compared to the end of the treatment session (r=0.513), and overall 90% of the paired measurments were in the zones A/B (clinically accurate or no risk from error) of the Clarke error grid. A recent study evaluating the Dexcom G6 CGM in patients with type 1 and type 2 diabetes during dialysis showed an overall MARD 13.8% (interquartile range 4.9-18.2%) when CGM was compared to matched SBGM measurements (99). In a meta-analysis of 524 routine dialysis patients with diabetes from 23 studies, Wang et al. confirmed a good correlation of CGM and BGM values (r=0.837) (100).

### Interpretation of CGM

The latest international guidelines on CGM for analysis and treatment goals were published in 2019 (36). “Time-in-range” describes the percentage of time spent with CGM reading between 70-180mg/dL/3.9-10.0 mmol/L). For most adults with diabetes, the consensus recommendations suggest that an appropriate goal is to achieve a percent time-in-range goal of over 70 percent, if achievable without undue risk of hypoglycemia (36, 101). Recommended treatment goals for patients with diabetes and comorbid conditions, such as chronic kidney disease, includes maintaining time-in-range >50% and time-below-range (<70 mg/dL3.9 mmol/L) <1% (36),. Several metrics exist for assessment of short-term, glucose variability (39), including the percent coefficient of variation (%CV), which is predictive of risk for severe hypoglycemia. In the past, CGM data were used to derive an “estimated A1c” calculated from mean glucose levels. However, in order to reduce confusion with measured HbA1c values, this predicted value of HbA1c is now referred to as a glucose management indicator (GMI) (31). CGM data indicate that the laboratory HbA1c may over-estimate the mean glucose in black persons compared to white persons (102).

### CGM clinical studies in the ESRD population

Clinical studies using CGM in the ESKD population have explored: (1) changes in glucose levels with hemodialysis; (2) comparison of CGM to standard glycemic markers in the setting of ESKD; and, (3) clinical benefit to be derived from use of CGM in diabetes management in the ESKD population.

(1) The advent of CGM has confirmed two known glycemic patterns in patients undergoing hemodialysis treatments. Several studies have demonstrated that glucose levels overall are lower on the day of the dialysis treatment. Using 24-hour CGM data, Kazempour-Ardebili et al. reported roughly 25% lower mean glucose values in type 2 patients during the day of dialysis (103). The average 24-hour glucose levels on nondialysis days was 38 to 187mg/dL (2.1 to 10.4 mmol/L) higher, compared to the average on dialysis days. The most common glucose nadir and risk of asymptomatic hypoglycemia was within 24 hours following dialysis. Euglycemic clamp data indicate a 25% reduction in basal insulin requirements the day after dialysis compared to the day before (62). In the meta-analysis by Wang et al, the average CGM values were significantly lower during dialysis in comparison with predialysis CGM values in patients with diabetes (MD: -2.11mmol/L or -40.3mg/dL, p=0.0003) (100). Divani et al. have shown using CGM in 36 diabetic hemodialysis patients the glucose variability as determined by coefficient of variation and hypoglycemia are higher on dialysis days (104).

(2) Several studies have evaluated the comparative clinical performance of CGM with HbA1c in diabetic patients with chronic kidney disease, including patients on hemodialysis. Two recent reports on predialysis patients have addressed the question of accuracy of long-term markers such as HbA1c, using its correlation with CGM as the primary outcome measure. Presswala evaluated CGM and HbA1c in CKD patients with type 2 diabetes and eGFR levels 78-45mL/min, using mean glucose levels over 14 days and mean HbA1c levels at the end of the CGM period. The Pearson correlation coefficient was 0.82, and the correlation was not affected by the severity of CKD (105). In a similar clinical study, Zelnick reported an overall Pearson coefficient of 0.78 (28). In contrast, a much weaker correlation in ESKD was reported by Riveline et al, who compared mean CGM values and hemoglobin A1c in dialysis and nondialysis patients with diabetes. The correlation between mean CGM and hemoglobin A1c values in hemodialysis patients was only 0.47, p=0.042. Poor metabolic control was identified using CGM data, which was not suspected based on HbA1c levels, even after correction for anemia (94).

The longitudinal association of glucose levels with the HbA1c was reported in 37 diabetic hemodialysis patients who used CGM over seven days to assess glycemic control (106). The area under ROC curve (AUC) for HbA1c to detect poor glycemic control was 0.776, indicating a fair association of HbA1c with average glucose levels. In the meta-analysis by Wang et al, the correlation coefficient between CGM and HbA1c in diabetes patients on dialysis (converting values from individual studies to Fisher’s z-scores) was =0.523 (100).

(3) To date there has been limited investigation on the potential utility of CGM in the management of diabetes in patients with ESKD. In the DIALYDAB pilot study reported by Joubert et al. (107), fifteen hemodialysis patients with diabetes used two different glucose measurements – SBGM (three per day) and CGM – each for a period of six weeks, to guide their insulin therapy. SBGM and CGM profiles were remotely analyzed by a diabetes specialist, who then provided therapeutic input to dialysis physicians. Use of CGM was associated with a greater number of alterations in the insulin regimen, improved glycemic control, and fewer episodes of hypoglycemia. Kepenekian et al. evaluated the use of CGM as a tool to guide treatment decisions in a multi-center study of 28 diabetic hemodialysis patients (108). Use of CGM allowed intensification of CGM-adapted insulin therapy over three months, with significant reductions in CGM-derived glucose levels and Hb A1c, despite no increase in symptomatic hypoglycemia. In a randomized crossover trial in adults with type 2 diabetes requiring dialysis, Boughton et al. have demonstrated that use closed loop insulin therapy - with an insulin pump, CGM and control algorithm to adjust insulin delivery is associated with improved time-in-range (3.9-10.0 mmol/L) and less hypoglycemia (109)

### Goals in the ESRD patient

**Figure 6:**
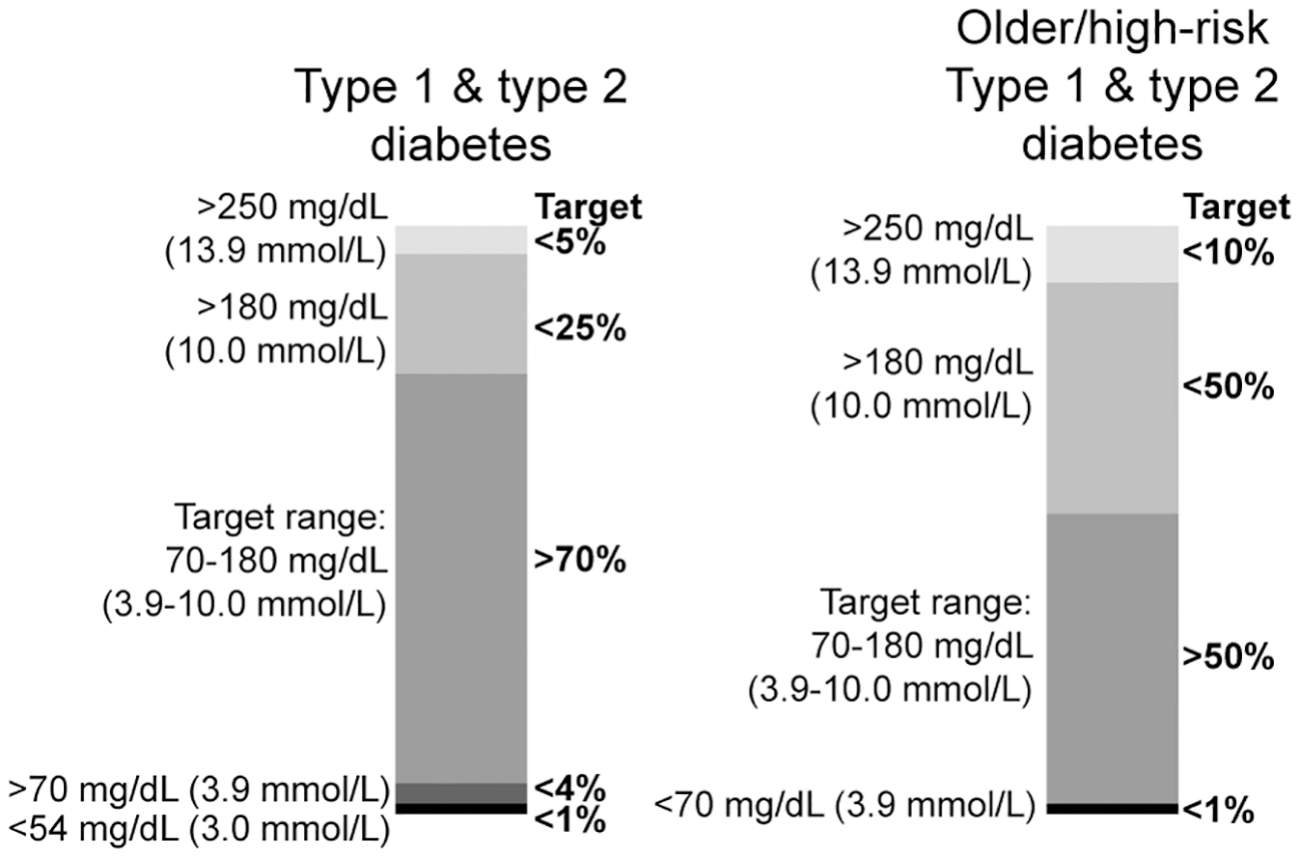
Consensus recommendations for CGM-based targets in different diabetes populations (see text). Modified with permission from Ref (36)..

Available guideline recommendations designed to address the use of CGM data to optimize clinical outcomes do not specifically include patients on dialysis (36). The consensus recommendations for lower risk patients with diabetes are shown in **Figure 6**. In comparison, it is recommended that patients with diabetes and higher risk due to comorbid conditions such as renal disease maintain time-in-range (70-180 mg/dL/3.9-10.0 mmol/L) >50% and time-below-range (<70 mg/dL/3.9 mmol/L) <1%, (**Figure 6**) (36).

### Current eligibility criteria

Despite the growing body of evidence supporting CGM use in type 1 diabetes and, more recently, the broader type 2 diabetes population, current eligibility criteria required by public and many private insurers are making access to this technology difficult for the ESKD diabetic population (36). There is a need for outcome studies demonstrating the potential benefit of CGM to provide the evidence base to support broader coverage of this technology by payers (110).

## Conclusion

Glucose management in ESKD patients with diabetes mellitus imposes challenges on both the patient and the dialysis provider. Evidence that current Hb A1c-based indicators of glycemic control adequately predict outcomes related to morbidity and mortality in diabetic ESRD patients remains insufficient. Closer glucose monitoring of glycemia would appear to be essential in ESKD because of various factors that impact glucose metabolism, related to abnormal glucose and insulin homeostasis. The more intensive and automated glucose monitoring facilitated by CGM technology may be of particular value in hemodialysis patients, insofar as hemodialysis exaggerates the state of glycemic variability. Until recently, CGM has been inadequately studied in the ESKD population. As a result, it is important that the CGM innovation be validated and its utility determined in the ESKD population. There is a need to better understand how CGM metrics can impact clinical outcomes in the hemodialysis population. In the meantime, CGM is an accurate diagnostic tool that should empower hemodialysis care providers to recognize and mitigate high-risk hypoglycemia and hyperglycemia in the context of the hemodialysis procedure itself.

## Author contributions

MW conceived of the review topic and was the primary author in the manuscript. Except for sections on glucose sensor technology, MW was the primary author. MW edited the entire manuscript. HW and DS are recognized experts in diabetes technology; they reviewed the content of the entire manuscript and were primary authors of the section(s) on glucose sensor technology. All authors contributed to the article and approved the submitted version.
